# Paraneoplastic syndrome mimicking adult-onset Still's disease caused by advanced lung cancer: a case report

**DOI:** 10.1186/1471-2407-11-487

**Published:** 2011-11-16

**Authors:** Ning Wu, Qiang Li, Chang-Xin Gu, Toqeer Ahmed, Xiao-Peng Yao

**Affiliations:** 1 Department of Respiratory Medicine, Changhai Hospital, Second Military Medical University, Shanghai, China; 2 Troops 95958 People's Liberation Army, Shanghai, China; 3 Department of General Medicine, Combined Military Hospital, Gilgit, Pakistan

## Abstract

**Background:**

Paraneoplastic syndromes (PNSs) are common complications of lung cancer and often develop preceding the diagnosis of primary malignancy. Rheumatologic PNSs mimicking Adult-Onset Still' s Disease (AOSD) is a rare condition with only a limited number of cases reported in the literature, none of which was associated with lung cancer. It is often difficult to differentiate AOSD-like paraneoplasia from coincidental AOSD based on the clinical manifestations.

**Case presentation:**

Here we present a 56-year-old man with advanced lung adenocarcinoma who developed a remittent fever together with pharyngodynia and joint pain after first cycle of chemotherapy with paclitaxel plus carboplatin. Although a leukocytosis was detected, no evidence of infection was acquired and empirical antibiotic treatment was ineffective. A temple skin rash, abnormal hepatic function and a remarkable elevated level of serum ferritin occurred later in this patient, which highly supported a potential diagnosis of AOSD. The patient was finally diagnosed as AOSD-like PNS considering the good and prompt response to a short-term administration of non-steroidal anti-inflammatory drug and subsequent cycles of effective chemotherapy with pemetrexed plus cisplatin.

**Discussion and conclusions:**

Though rare, AOSD-like PNS can be one of the potential diagnoses in lung cancer patients with fever of undetermined origin, especially those having no response to antibiotic treatment. Management consists of control of the underlying malignancy and symptomatic treatment of the syndromes with non-steroidal anti-inflammatory drugs or corticosteroids.

## Background

Paraneoplastic syndromes (PNSs) are defined as signs or symptoms that occur as a result of organ or tissue damage at locations remote from the primary tumor site or metastases. Lung cancer is one of the most common malignancies known to be predisposed to PNSs, most frequently associated with advanced disease [1,2]. The types of PNSs vary substantially and often develop preceding the diagnosis of primary malignancy [3]. For most PNSs, the best treatment is to treat the underlying malignancy [4]. Here we report a rare case of PNS developed in a late-stage lung cancer patient after one cycle of chemotherapy, which closely resembled the typical manifestations of Adult-Onset Still' s Disease (AOSD).

Written informed consent was obtained from the patient for publication of this case report.

## Case presentation

### Medical history

A 56-year-old Chinese man with a two-month history of cough and hemoptysis was admitted to our hospital in September 2010. He was a nonsmoker and had no remarkable past medical history. Lung adenocarcinoma of left lower lobe with cerebral metastasis was diagnosed by bronchoscopic biopsy and imaging.

Chemotherapy with paclitaxel (135 mg/m^2^) plus carboplatin (AUC 6 mg/ml·min) was administered on September 15, 2010 and the patient was discharged 2 days later.

A routine blood analysis after a week showed a moderately decreased white cell count (WCC) of 2,660/ul with 67% neutrophils. No specific treatment was given because of the absence of symptoms. The patient visited a local hospital on October 2, 2010 because of a sore throat and mild fever. Blood analysis showed a WCC of 3,440/ul with 67% neutrophils and a platelet count of 7.4 × 10^4^/ul. Although prophylactic antibiotic was administered, the pharyngodynia continued and a spiking fever was observed 4 days later complicating with joint pain involving bilateral knees and shoulders. The patient was referred to our department on October 10, 2010.

### Biochemical and physical/clinical examinations

Physical examination on admission showed remittent fever of up to 39.6°C, peaking in the post meridiem; decreased breath sounds in the left lung, which may be due to the primary tumor. The superficial lymph nodes were not palpable. Arthritis was not apparent until the body temperature rose. Peripheral blood tests showed a leukocytosis of 13,470/ul with 87% neutrophils, and a platelet count of 7.2 × 10^4^/ul. The C-reactive protein was 88.1 mg/l and erythrocyte sedimentation rate 22 mm/H.

Considering the history of chemotherapy for lung cancer and previous leukopenia of this patient, a latent infective disease was suspected and intravenous Moxifloxacin (0.4 g/day) and Cefepime Hydrochloride (2 g/12 h) were administered for 5 days. However, no improvement of the symptoms was achieved. Evanescent maculopapular rash appeared on the right side of his back on the fifth hospital day. Blood analysis showed an increased level of WCC (15,400/ul) with 87% neutrophils and an abnormal hepatic function without hepatosplenomegaly, including elevated alanine aminotransferase (213 IU/l), aspartate aminotransferase (128 IU/l) and alkaline phosphatase (309 IU/l). The rheumatoid factor and antinuclear antibodies were both negative. No infectious agent was isolated from the blood, sputum, pharynx swab or urine cultures performed. Abdominal ultrasonography and electrocardiogram were normal. Neither a contrast-enhanced chest computed tomography (CT) scan nor a position emission tomography-CT scan found any infective or inflammatory sign except the tumors in lung and brain which were of the same size as previously detected. So the antibiotic treatment was stopped. An injection of 40 mg methylprednisolone was administered. The body temperature turned normal temporarily and increased to 39°C again about 40 h later. A remarkable elevated level of serum ferritin (2,000 ng/ml, reference range 13 ~ 200 ng/ml) was noticed.

### Treatment and outcome

Based on these findings, a potential diagnosis of AOSD was strongly proposed. Then oral nimesulide (100 mg/12 h), a neotype of non-steroidal anti-inflammatory drug (NSAID), was started and the temperature decreased gradually to normal in 5 days, together with improvement of other symptoms. The levels of both WCC and transaminases of peripheral blood returned to normal range. The performance status of the patient was notably improved. So another cycle of chemotherapy with pemetrexed (500 mg/m^2^) plus cisplatin (75 mg/m^2^) was performed on October 29, 2010 without any apparent adverse effect observed.

Treatment with nimesulide was continued for about 3 weeks with no fever or any other symptom recurrence afterwards. Then nimesulide was stopped and a second cycle of pemetrexed-cisplatin chemotherapy was administered. A repeat CT scan after two cycles demonstrated a significant reduction in size of the lung mass. Routine peripheral blood tests and liver function tests stayed normal. Rheumatoid factor and antinuclear antibodies were negative all along. Repetitive blood tests showed rapid reduction of serum ferritin levels (483.7 ng/ml and 394 ng/ml after the first and second cycle of chemotherapy with pemetrexed plus cisplatin, respectively). Four more cycles of chemotherapy with pemetrexed plus cisplatin were then administered every 21 days with good tolerance achieving partial remission of disease. No AOSD-like manifestation occurred till now. Although a prompt clinical response to NSAID was achieved with resolution of fever, a diagnosis of PNS mimicking AOSD was finally made considering the persistent remission of the clinical manifestations to the effective chemotherapy.

## Discussion and conclusions

Non-small cell lung cancer (NSCLC) is the leading cause of death related to cancer worldwide [5]. Cytotoxic chemotherapy remains the mainstay of treatment for patients with metastatic NSCLC [6]. Fever is a common complication in advanced lung cancer patients receiving chemotherapy. Secondary infection should be excluded first because of the high incidence of granulocytopenia in these patients. The other common reasons of cancer-related fever include tumor necrosis, progressive disease, disturbance of central nervous system because of metastasis, and adverse effects of some cytotoxic drugs. In rare cases, fever can be caused by PNSs or some febrile diseases concomitant with cancer. Here, we present a 56-year-old man with advanced lung adenocarcinoma who developed a remittent fever together with pharyngodynia and joint pain after first cycle of chemotherapy. Although a leukocytosis was detected, no evidence of infection was acquired and empirical antibiotic treatment was ineffective. A temple skin rash, abnormal hepatic function and a remarkable elevated level of serum ferritin occurred later in this patient, which highly supported a potential diagnosis of AOSD. The patient was finally diagnosed as AOSD-like PNS considering the good and prompt response to a short-term administration of NSAID and subsequent cycles of effective chemotherapy.

AOSD is a systemic inflammatory disease with unknown etiology, characterized by high spiking fever, evanescent salmon pink rash, arthritis, and leukocytosis with neutrophilia. Sore throat, liver dysfunction, lymphadenopathy and hepatosplenomegaly are also common clinical findings. Hyperferritinemia may assist in establishing the diagnosis and correlates with disease activity [7-9]. The Yamaguchi criteria for AOSD classification is widely used for diagnosis [10]. However, making a diagnosis of AOSD necessitates excluding the other etiologies of febrile conditions due to infection, malignancy, and other rheumatic diseases. Several cases of malignancy with typical manifestations of AOSD have been reported in the literature, including malignant lymphoma, myeloproliferative disorders, and some solid cancers (such as breast, thyroid and esophagus) [8,11-17]. Rheumatologic PNSs mimicking AOSD were finally diagnosed in some of these reported cases. However, it is difficult to differentiate AOSD-like paraneoplasia from coincidental AOSD based on the clinical manifestations. Symptoms of PNSs often precede the diagnosis of a neoplasm and resolve after effective anticancer treatment. Ahn JK [18] has reported a case of AOSD diagnosed concomitantly with occult papillary thyroid cancer, in which the clinical symptoms improved promptly after a high dose corticosteroid treatment before thyroidectomy and radioiodine therapy was performed. A Japanese case of chronic myelogenous leukemia diagnosed 2 years after the onset of AOSD was reported by Nakagawa Y, indicating that an intrinsic relationship may exist between AOSD and some malignancy [19].

As to the present case, whether AOSD was a part of PNSs or a coincidental disease was difficult to determine at first. Along with the remission of cancer after subsequent cycles of effective chemotherapy, a rapid and persistent improvement of clinical abnormalities was achieved, which finally enabled us to make a diagnosis of PNS that resemble AOSD. To our knowledge, this is the first report of PNS mimicking AOSD caused by lung cancer. Bosch-Barrera J [20] has described a clinical vignette of a metastatic NSCLC patient compatible with the development of an AOSD after the administration of the first cycle of pemetrexed-gemcitabine regimen, which was ascribed to potential adverse effects of chemotherapy regimen though no direct causal relation to them had been established. In our patient, the AOSD-like manifestations developed after the first cycle of chemotherapy with paclitaxel-carboplatin and disappeared along with the remission of the tumor after the effective chemotherapy with pemetrexed-cisplatin. No similar clinical phenomenon associated with either of these two cytotoxic drugs has been reported in the literature. Though adverse drug reaction cannot be completely ruled out, we do not think there was any direct association between the occurrence of rheumatologic symptoms and the anticancer regimen in this case.

In summary, we described a case of PNS caused by a lung adenocarcinoma after one cycle of chemotherapy which was nearly indistinguishable from AOSD. Though rare, AOSD-like PNS can be one of the potential diagnoses in lung cancer patients with fever of undetermined origin, especially those having no response to antibiotic treatment. For these patients, treatment of the underlying tumor is the best therapy.

## Competing interests

The authors declare that they have no competing interests.

## Authors' contributions

NW: reviewed the literature, drafted and edited the manuscript; QL: aided in acquisition and interpretation of the data; CXG: and TA: carried out the literature search, reviewed the literature and helped in editing the manuscript; XPY: involved in the final revision of the manuscript and coordinated the submission. All authors were involved in the patient active management. All authors read and approved the final manuscript.

## Pre-publication history

The pre-publication history for this paper can be accessed here:

http://www.biomedcentral.com/1471-2407/11/487/prepub
